# Validation of a Script to Facilitate Social Determinant of Health Conversations with Adolescent Patients

**DOI:** 10.3390/ijerph192214810

**Published:** 2022-11-10

**Authors:** Emily M. Giorgi, Matthew J. Drescher, Zachary K. Winkelmann, Lindsey E. Eberman

**Affiliations:** 1 Department of Applied Medicine and Rehabilitation, Indiana State University, Terre Haute, IN 47803, USA; 2 Department of Exercise Science, University of South Carolina, Columbia, SC 29208, USA

**Keywords:** focused history script, cultural proficiency, patient-centered care, patient interview

## Abstract

Current social determinants of health (SDOH) tools exist to assess patient exposure; however, healthcare providers for the adolescent population are unsure of how to integrate SDOH knowledge into clinical practice. The purpose of this study was to validate a focused history script designed to facilitate SDOH conversations between clinicians and adolescents through the use of the Delphi method. Six individuals (1 clinician, 5 educators/researchers) participated as expert panelists. Panelists provided critical feedback on the script for rounds 1 and 2. For rounds 3–7, panelists received an electronic questionnaire asking them to indicate agreement on a 6-point Likert scale (1 = strongly disagree, 6 = strongly agree). We defined consensus as mean item agreement ≥ 5.0 and percent agreement ≥ 80%. In round 7, panelists rated overall script level of agreement. After seven rounds of feedback, the focused history script achieved content validity with 100% of panelists agreeing on the final 40-item script. A focused history script for the SDOH was content validated to aid conversations between healthcare providers and adolescent patients on factors that affect their life, school, and play. Addressing social determinants of health with adolescent patients will improve cultural proficiency and family-centered care delivered by school healthcare professionals.

## 1. Introduction 

The social determinants of health (SDOH) are the various conditions of an individual’s environment affecting the ways in which they live, work, learn, and play [1], and in doing so have a significant role in patients’ health. The United States Department of Health and Human Services categorizes SDOH into 5 separate domains: economic stability, education access and quality, healthcare access and quality, neighborhoods and built environments, and social and community context [2]. These SDOH can either positively or negatively influence patient health outcomes depending on a variety of factors; however it is the negative impacts of SDOH that contribute to the decreased likelihood of optimal patient health [3,4]. Higher exposure to negative influences of SDOH leads to an individual’s increased susceptibility to a variety of health conditions, with minority populations having an increased exposure [5]. For instance, individuals from a low socioeconomic background are more susceptible to health morbidities, such as cancer, diabetes, heart disease, and an overall decrease life-expectancy [6]. As much as 70% of preventable deaths in the United States can be attributed to either previous or current exposure to various SDOH [7]. Additionally, SDOH affects every stage of life, from early childhood, adolescence, and adulthood, affecting both immediate and long-term health [8].

Health systems are responsible for responding “appropriately to the different needs of different social groups, and to take the lead in encouraging a wider and more strategic approach” [8] at all public health levels to ultimately address SDOH within adolescent patients. However, addressing SDOH is often negatively affected by health inequity. Health inequity represents the “unfair, systematic differences in health and health outcomes,” and is the result of poorly structured public health policies [9]. Public health initiatives, particularly at local levels (including county and state government levels), are intended to mitigate the adverse health outcomes resulting from the SDOH on disadvantaged communities, especially adolescent populations [8]. Public health intervention strategies to address SDOH within the adolescent population are most effective through multi-sector approaches, like school-based health centers (SBHC) [10,11]. SBHCs have been shown to decrease various healthcare access barriers due to the nature of the health care provided often being dependent on location [10,12]. As patients are not being seen in a private office, barriers such as transportation issues, lack of insurance, and guardians taking time off work for their child’s appointments are eliminated [12]. In a SBHC, providers are in the unique position to integrate various preventative healthcare strategies, including addressing SDOH, with clinical care, as the school setting provides close proximity healthcare combined with the development of strong clinician-patient relationships [10]. In the secondary school setting, school-based healthcare providers often consist of athletic trainers and school nurses, who frequently work with social workers to provide care for adolescent patients. These providers are well placed to advocate for the well-being of their patients, however, without a comprehensive understanding of the various external factors affecting overall patient health, this is not achievable. 

To provide optimal care to adolescent patients and to gain this essential comprehensive understanding of the SDOH factors affecting patient health, clinicians must be able to demonstrate culturally proficient behaviors and address SDOH with patients. Cultural proficiency is defined as the “ability to effectively collaborate with individuals of different cultures, and such [proficiency] can help improve healthcare experience and outcomes” [5]. Due to the various health inequities caused by flaws within public health systems, minoritized populations are often more likely to have an adverse health outcomes caused by SDOH, as they are the result of a poorly structured public health framework [13]. A healthcare provider’s cultural proficiency is essential to addressing SDOH with patients effectively. Yet, there is a disconnect between the plethora of SDOH tools already established [14] and the clinician’s ability to integrate these tools into clinical practice, thus contributing to low levels of cultural proficiency. Previous literature surrounding cultural proficiency in a variety of healthcare professions suggests clinician perception of cultural proficiency is significantly higher than actual behaviors displayed in clinical practice [15,16,17,18]. Furthermore, research shows that only about half of the healthcare community receives training related to cultural proficiency; however, as much as 80% of clinicians describe themselves as being satisfied with their perceived cultural proficiency [19]. When clinicians implement culturally proficient behaviors, marginalized and disadvantaged patients feel safe and are empowered to discuss how health inequities are negatively affecting their health, thus giving patients more control over their health [8,20]. The ability of clinicians to effectively respond to the individualized cultural needs of patients can lead to improved patient outcomes, according to current research [21,22,23].

Cultural proficiency levels of healthcare professionals is not the only factor influencing a clinicians likelihood of addressing SDOH with patients. Recent studies have explored the influence of educating healthcare professionals on the importance of assessing SDOH and found that with an increased knowledge of SDOH, providers are more inclined to screen patients for SDOH exposure [24,25,26,27]. Clinicians in the secondary school setting found themselves unsure how to sensitively and successfully discuss SDOH factors with their adolescent patient population [24,25]. Furthermore, adolescent primary care specialists recognize it is their responsibility to address SDOH with patients, however, they also described discomfort in addressing SDOH domains outside of socioeconomic concerns [28]. 

Many screening tools already exist to assess SDOH domains, ranging from assessing a singular SDOH to more comprehensive, all-SDOH-domain-encompassing tools [29,30,31,32]. However, a common concern of healthcare professionals is that SDOH topics may be uncomfortable for patients to discuss. Some clinicians have expressed not wanting to jeopardize rapport and patient relationships when seeking out this information due to the sensitive nature of SDOH [33]. In contrast, other clinicians are comfortable with the material yet feel as though they lack a way to initiate SDOH conversations with patients [34]. To our knowledge, only one tool, WE CARE, has been developed to initiate SDOH conversations with the adolescent population [35]. While transformative in nature as it was the first tool written specific to the adolescent population, it does not cover the full spectrum of SDOH, as it fails to address all five domains, and limits patient responses to the same three multiple-choice answers for each question. To date, there are no comprehensive tools developed to initiate these conversations with adolescents [8]. In order to improve clinician confidence regarding addressing SDOH, a validated and comprehensive focused history script for clinicians working with the adolescent population to complement their existing SDOH knowledge and desire to screen patients is needed. This script would act as a guide to initiate SDOH conversations, and used in conjunction with pre-existing SDOH assessment tools, rather than taking the place of screening tools. This tool would benefit any healthcare clinician providing care for adolescent patients, regardless of a particular setting. Providers within SBHCs in particular would benefit greatly from the use of this script, due to the close rapport and proximity these providers have with their adolescent patients. By implementing this script into clinical practice, clinicians will bridge the gap from initial SDOH conversation to implementation of SDOH screening tools. Therefore, the purpose of this research is to establish content validity for a comprehensive, open-ended format, focused history script designed to facilitate SDOH conversations between clinicians and adolescents.

## 2. Materials and Methods

The research team used the Delphi panel technique to develop the focus history script’s content validity. The Delphi panel technique is commonly used in medical fields in which experts develop group consensus on content through multiple rounds of communication with researchers [36,37]. While the process takes approximately three to five rounds to reach validation, the process continues for as many rounds necessary to achieve consensus between panelists [38]. The Delphi technique is the preferred technique for establishing content validity for tools and guidelines within various healthcare settings and allows for each expert to provide both individual and specific feedback, ensuring multiple perspectives are considered when developing consensus [38]. Content validity is crucial for this script, as it ensures the elements of a developed instrument is “relevant to and representative of a targeted construct for a particular assessment purpose” [39]. As this script centers around the specific target population of adolescents, establishing content validity is essential to ensuring script questions are appropriately designed for clinicians to get valuable SDOH information about, and from, their specific patient population.

Recruitment of potential panelists occurred via email, which included a brief overview of the Delphi process and aim of the proposed script. A Delphi panel does not have a required number of panelists, therefore size of the panel varies based on both the project’s purpose and timeframe [38]. Literature suggests a minimum of three panelists, with five panelists providing sufficient control of chance agreement [40]. The research team selected a panel of 6 experts (5 researchers, 1 clinical athletic trainer) who all have an extensive background and understanding of SDOH in a healthcare setting. Diversity in healthcare settings and professions allows for script assessment through different perspectives on patient care. Panelists’ years of experience within healthcare ranged from 4.5–23 years with varied settings and fields represented (Table 1). Qualifications pertaining specifically to SDOH topics within the Delphi panelists include health equity within marginalized populations, geographical context affecting health, and culture and diversity in health care. Additionally, recent research published from selected experts range SDOH topics, such as rural health, psychosocial determinants of health, athletic training education of SDOH, medically underserved populations, etc. 

The research team developed a preliminary list of history questions to facilitate SDOH conversations with patients. The questions were developed from SDOH domains, as established by the Center for Disease Control and Prevention and the United States Department of Health and Human Services [41]. The script was designed to be versatile; it can to be used as either a clinician-led one-on-one conversation with the patient, or the patient can complete the script alone, allowing the clinician to review and address any potential concerns later. The tool was designed and validated specifically for the adolescent population, including primarily open-ended questions, allowing patients to provide information that may be filtered out with traditional yes/no questioning, typical of available screening tools. The script was not designed to assess SDOH exposure of a patient, but rather to act as a guide for SDOH conversations to occur between clinicians and adolescent patients. 

**Figure 1:**
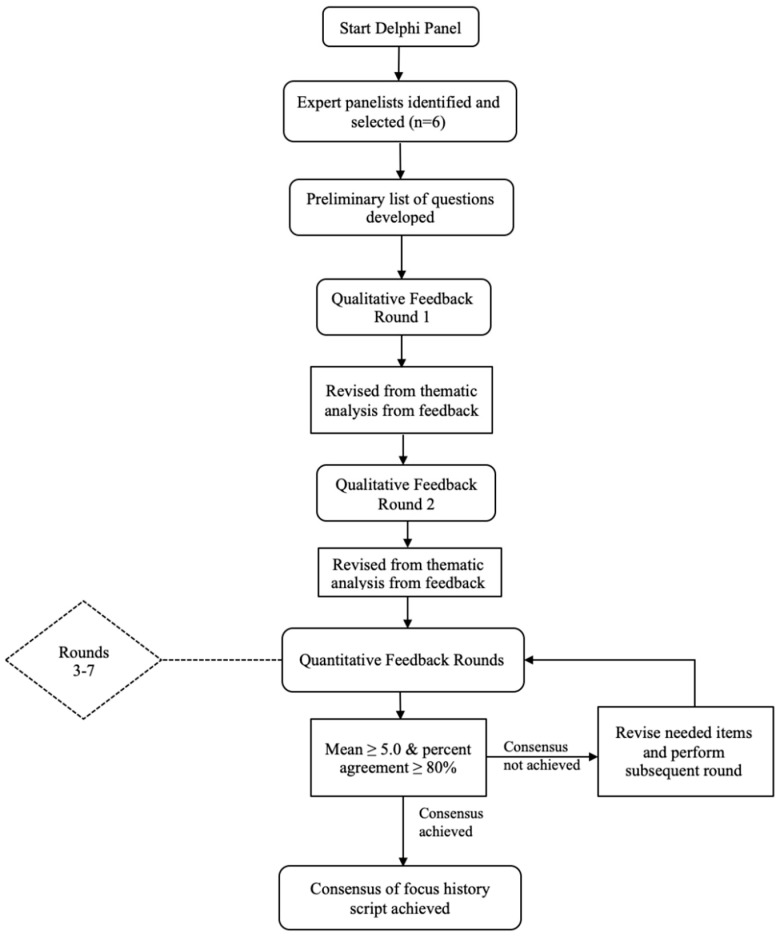
Delphi Panel Process.

As mentioned previously, while the Delphi panel technique uses approximately 3 to 5 rounds of review to develop consensus, the process continued for as many rounds necessary to achieve this benchmark [40]. The first two rounds focused on qualitative, written feedback from expert panelists, whereas rounds 3 through 7 used quantitative metrics from a six-point Likert scale measuring agreement levels of script item relevancy to current literature, as well as item inclusion into the script (Figure 1). We used mean, standard deviation, and percent agreement of panelists to guide subsequent rounds of review. The process spanned 8 months (March–October 2021). 

Delphi round 1. In round 1, panelists received the original script in a word document via email. We instructed panelists to use track changes in Microsoft^®^ Word (version 16.16.27, Redmond, WA, USA) to indicate if an item in the script should be excluded, refocused, or added into the tool. Panelists were asked to provide feedback within 1 week. We inductively coded the feedback for common themes and used those common themes to make changes to the script. Revisions based on analysis of the feedback from this initial round took approximately 1 week. 

Delphi round 2. In round 2, panelists received a revised script based on feedback from round 1 in a Microsoft^®^ Word document via email. Similar to round 1, we instructed panelists to use track changes in Microsoft^®^ Word to identify if an item should be revised in any capacity. Panelists were asked to provide feedback within two weeks for round 2. Revisions based on analysis of the feedback from this round took approximately two weeks. 

Delphi rounds 3–6. In rounds 3 through 6, we sent panelists the revised script in each round with an online questionnaire delivered via Qualtrics^®^ (Provo, UT, USA) asking the panelist to rate their levels of agreement on (1) reflection of current literature and (2) inclusion into the focused history script via a six-point Likert scale with extreme indicators 1 = strongly disagree and 6 = strongly agree for each of the script items. Additionally, we provided panelists space at the end of the questionnaire to provide open-ended feedback and comments on the overall script for further revisions if desired. Panelists were provided two weeks for each round of review and feedback. Analysis and revisions from each round required up to two weeks to complete. The analysis consisted of assessing each item’s mean scores and calculating the percentage agreement of all panelists. For consensus to be achieved, we required mean scores equal to or above a score of 5.0, and 80% or greater percent agreement. Revisions were drafted on items not achieving the validation benchmarks and then re-evaluated in the following round [40]. The same procedures were used in Rounds 4, 5, and 6; however, in round 5, we included citations to support inclusion of remaining items to show panelists what sources were being used to support inclusion of the remaining items. 

Delphi round 7. Prior to drafting the script for Round 7, the primary investigator consulted with panelists who were not yet scoring items relevant for inclusion to better understand the source of potential discrepancy. Consultation served as an alternative to the open-ended space to provide feedback and offered additional insight on how to better revise the remaining items needing validation. Once the research team achieved further clarity on the remaining items, the panelists received the following: a revised script from round 6, an updated questionnaire, and a summary of the findings from the most recent round. In addition, researchers gave panelists the same instructions to rate the level of agreement on reflection of current literature and script inclusion. Panelists were provided two weeks to provide feedback. Analysis of this final round required one day to complete. 

For the first two rounds of review, we inductively coded feedback to identify common themes and made revisions on common themes based on the open-ended feedback and Microsoft^®^ Word track changes on the proposed script from expert panelists. For subsequent rounds, the research team used the validation benchmarks to validate each item (mean ≥ 5.0 and a percentage agreement of ≥80%). As an item achieved the benchmark level of agreement, it was no longer included in future rounds and demonstrated content validation and consensus on that item [42]. Common percent agreement for Delphi studies within the healthcare setting range from 75–80%, thus the decision to use 80% as the established criteria for determining item validation [43,44]. Beginning with round 3 and upon conclusion of each round thereafter, we analyzed descriptive statistics on each item still needing validation, calculating mean, standard deviation, and percent agreement for item relevance to current literature and item inclusion. Calculations were performed using Microsoft^®^ Excel (version 16.16.27, Redmond, WA, USA) formula functions. 

## 3. Results

### 3.1. Round 1

Based on the feedback from round 1, panelist feedback on the proposed script consisted of removing two items, adding 14 items, and revising the remaining 23 items in the instrument. We gathered panelist feedback and revised the script as needed into a 37-item script. 

### 3.2. Round 2 

In round 2, panelists added 5 items and requested revisions of the original 37 items proposed in the revised script. We compiled panelist suggestions and revised the instrument accordingly into an updated 42-item script. 

### 3.3. Round 3

In round 3, panelists accepted 17 of the 42 script items (40.5%), rejected 2 items, and requested revisions of the remaining items (Appendix A Table A1). We used both the panelist quantitative and qualitative feedback from the questionnaire to further develop the instrument. 

### 3.4. Round 4

In round 4, panelists accepted an additional 10 items (43.5%), rejected 2 items, and suggested further modification to 11 of the proposed items (Table A1). We revised the remaining items accordingly based on panelist feedback.

### 3.5. Round 5

When we asked the panelists to rate their level of agreement of the updated 12 items in round 5, 3 of the 12 were accepted (25%), with 1 item added and 1 item removed (Table A1). Additionally, panelists suggested revisions of the remaining items. We used panelist comprehensive feedback to further develop the remaining script items. 

### 3.6. Round 6

In round 6, panelists rated their level of agreement of the remaining 8 items and accepted 4 of the 8 proposed items (50%). Furthermore, panelists recommended adding two additional items and making further revisions to the remaining items (Table A1). We used panelist quantitative and qualitative feedback, including a consultation for additional clarification, to further revise the remaining items. 

### 3.7. Round 7

Because all items met the validation benchmarks (Table A1), all remaining items were accepted into the SDOH script (Table 2). Furthermore, 100% of the panelists (6 of 6) strongly agreed with the content of the final version of the instrument. The content validated focused history script has a Flesch-Kincaid readability grade level of 5.1, suitable for adolescent patients. The final tool contained 11 items focused on healthcare access & quality, 4 items on education access & quality, 6 items on economic stability, 8 items on neighborhood & built environment, and 11 items on social & community context. 

## 4. Discussion

The purpose of this study was to establish content validity of a focused history script designed to facilitate SDOH conversations between clinicians and adolescents. We established content validity through expert consensus from seven rounds of Delphi panelist review. The script will require further investigation regarding reliability, training (if needed), and clinical usefulness. 

### 4.1. SDOH Screening 

Addressing SDOH in various healthcare systems is a “population-wide goal: like the goal of improving health, it includes everyone” [8]. However, previous assessment and screening tools surrounding SDOH are written for the general adult population [29,45], therefore frequently overlooking how the adolescent population is affected by negative SDOH. Adolescent patients exposed early in life to adverse SDOH are at an increased risk of sustaining a variety of long-term health consequences [31,46,47]. The literature surrounding health disparities suggests addressing SDOH improves health outcomes in patients [3,48]. All healthcare professionals providing care for the adolescent population have the opportunity and responsibility to address SDOH to mitigate health morbidities from developing later in life. Specifically, SBHCs aid in further mitigating the adverse effects of SDOH, as they place healthcare providers directly within the adolescent patient environment, overcoming various barriers that may be present in a non-community or school-based health system [49]. The frequent patient encounters by healthcare providers with adolescent patients allows for continual care addressing SDOH [50]. Even if the child does not have access to comprehensive healthcare, providers through SBHC such as school nurses and athletic trainers can serve as a primary access point to healthcare for these patients [3]. They are able to foster a trusting, professional relationship with patients, acting as an advocate for the patient against various healthcare disparities and inequities [3]. While this script is not intended for the sole use of healthcare providers within SBHCs, these providers are in a unique position in which they can directly improve patient outcomes through addressing SDOH with their adolescent patients, demonstrating culturally proficient behaviors through script implementation. 

These culturally proficient behaviors help build relationships while simultaneously empowering patients [51]. These relationships are essential to promoting health equity, as these “micro-level” relationships between the school staff and the community have the potential to develop into more “macro-level” structures, provoking entire healthcare system changes to aid disadvantaged populations [8]. A study performed in 2015 identified that while healthcare professionals recognize the need and importance of addressing SDOH in the pediatric setting, there are not many tools available to help bring these behaviors to fruition [52]. Additionally, there is no clear consensus on a single screening tool or time constraints to perform comprehensive SDOH screenings [53]. 

Although screening and assessment instruments for SDOH have existed for several years, and while they are often used on adolescents, these previous tools are not designed specifically for the adolescent population. Additionally, most of the current tools focus on economic stability assessment, followed by social and community context assessment [54]. In this study, the Delphi panel helped to establish a comprehensive, focused history guide for conversations between clinicians and adolescents, addressing barriers previously identified in the literature [8,24,25]. The following pre-existing SDOH assessment tools discussed are some the more effective tools in the literature to date, however all consist of commonly seen barriers regarding addressing SDOH within the adolescent population. For instance, the PRAPARE screening tool was developed to help clinicians both address and document SDOH risk factors present in patients [51], and consists of 21 total questions, with answer options limited to “yes,” “no,” or “I choose not to answer this question” [55]. As PRAPARE was developed with the goal of promoting SDOH documentation within electronic medical records (EMR) [32,55], the closed-ended nature of the questions in the screening tool, while efficient for EMR documentation purposes, does not promote conversation between clinician and patient. Furthermore, while PRAPARE has been the foundation for many other SDOH assessment tools as it was the first screening tool to ask questions pertaining to all SDOH domains, the questions are not sensitive to the adolescent population. Other well-known SDOH assessment tools designed to expand on PRAPARE are the AAFP Social Needs Screening Tool as well as the Health-Related Social Needs Screening Tool [32]. The AAFP Social Needs Screening Tool consists of 11 questions that address all five SDOH domains [30]. Each question has multiple choice response options, and patients select the option they identify most with. There is no overall score with the AAFP Screening Tool. However, each question has specific response items underlined, and if selected by the patient, indicates the patient needs additional resources or assistance within that particular SDOH domain [30]. While this bridges the gap from asking a question to providing follow-up assistance for patients, the multiple choice structure forces patients to generalize their SDOH experience without allowing them to elaborate on their selection, which prevents them from receiving the individualized assistance they may need. The Health Related Social Needs Screening Tool is very similar to the AAFP Screening Tool in regard to patient response options and clinician assessment, however, is more comprehensive in nature, as it has 26 total questions [29]. While all three of these relatively well-known tools just discussed have questions pertaining to each SDOH domain, none are specific to the adolescent population, limiting the patient populations benefitting from PRAPARE, AAFP Screening Tool, and the Health Related Social Needs Screening Tool. Our script takes a comprehensive approach towards facilitating conversations with the adolescent population. Unlike pre-existing SDOH screening tools, this script does not score patient responses based on risk exposure; rather, it serves as a conversation guide for clinicians through the use of primarily open-ended questions to identify SDOH topics that may require additional exploration with the patient. Because of this intentional structuring of the script, it is not to take the place of screening tools. Combining our script with previously existing SDOH assessment tools, clinicians providing care for adolescent patients in any setting will be able to seamlessly make the transition between initiating SDOH conversations, to assessing the extent to which a patient is affected adversely by various SDOH factors, ultimately allowing clinicians to streamline more effective and optimal care plans based on each patient’s individual needs. 

Through the consensus of expert panelists, we established a valid focused history script consisting of 40 items. Each domain consists of at least four items, ensuring a well-rounded instrument. All items and constructs achieved consensus at or above the validation benchmarks. Panelists struggled to achieve consensus in rounds 3 to 6 regarding the SDOH factors of patient access to healthcare and economic stability, likely due to the amount of screening tools already developed to assess these particular SDOH domains. In feedback, the panelists remarked that while the inclusion of the items related to these SDOH is crucial, we needed to refine the specific questions to probe patients effectively. This feedback aligns with pre-existing tools regarding these SDOH domains relying heavily on close-ended questions to score patient responses [54,55]. Because the emphasis is to guide conversation rather than scoring patient responses, the open-ended questions presented new challenges to the panelists, as this question structure has not been used on previous SDOH tools. As previously stated, to our knowledge, the WE CARE survey is the only other tool developed for the adolescent patient population to encourage SDOH discussion, developed with the intention of connecting SDOH screening with appropriate referrals [35,56]. However, WE CARE limits patient responses to “yes,” “no,” or “maybe later,” and only consists of 11 questions, making it difficult to gain a comprehensive understanding of patient SDOH exposure. 

Due to the nature of adolescent healthcare, implementation of this script specifically within SBHC environments would be most effective upon annual check-ups, such as during annual pre-athletics participation examination screenings of student-athletes. Screenings upon initial and routine visits allow for initial patient needs to be met and monitored, as needs often change over time [31]. These routine screenings allow for the ability to detect change in returning patients, and are often the focus of initial and standard interactions between clinicians and patients, aligning with principles established regarding effective SDOH screening. 

### 4.2. Implications for School Health Policy, Practice, and Equity

With 40-items included in the script, the implementation may be time-consuming for clinicians. Additional time burdens and resistance to change are commonly cited reasons for the lack of instrument implementation [57,58,59]. If these barriers persist in using this tool, cultural proficiency levels will remain low due to lack of script implementation. Across various healthcare fields, the implementation of screening and assessment tools has proven to be an ongoing challenge [60,61,62,63]. To combat this script being yet another tool within SDOH resources that is seldom used, and to encourage substantial and more permanent behavior change, clinicians should refer to the following considerations towards effective script implementation and long term healthcare provider behavior change. These recommendations offer suggestions regarding the appropriate timing of implementation and effective forms of training healthcare professionals on how to incorporate the developed script into regular clinical practice.

### 4.3. Ensuring Script Implementation

Healthcare professionals of all experience levels should implement this validated script to provide optimal patient care and improve cultural proficiency behaviors. Prior to the autonomous practice, we suggest script implementation be individual based, particularly in healthcare educational programs. Education programs have a responsibility to equip future clinicians with the knowledge and skills needed to provide optimal and culturally proficient patient care. SDOH curriculums have succeeded through interactive instruction, simulations, and allowing students to participate in service learning opportunities [27,64,65]. Providing students with hands-on learning experiences and the ability to practice using this focus history script in professional healthcare education programs will empower students with the confidence to initiate these sensitive conversations effectively and will empower their adolescent patients to advocate for themselves with their healthcare provider and further improve their health [8,20]. As the student engages with the curriculum, learner confidence will rise, increasing the likelihood of addressing SDOH through script implementation and improving culturally proficient behaviors upon entry into autonomous practice [4,65]. Upon clinician entry into practice, SBHCs play an integral role in the continual professional development of their clinicians to actively work towards decreasing health inequities. To create lasting change, the clinician should transition from individualized script implementation to expanding script implementation into interprofessional care teams throughout SBHCs through professional development opportunities. Previous literature identified lack of effective workflows and continuous patient care as a key challenge to effectively addressing SDOH in SBHCs [11]. Therefore, engaging in interprofessional practice when using the script may mitigate this difficulty, simultaneously stimulating lasting change and progress in working towards health equity. Involving all individuals within SBHCs who regularly advocate for improving adolescent health outcomes (e.g., teachers, social workers, and healthcare providers) allows for a seamless transition between initiating SDOH conversations and providing patients with any necessary resources, ultimately leading to a decrease in adolescent health disparities [20]. When developing professional development programs for interprofessional teams, we suggest scaffolding, the process of implementing a variety of educational opportunities to build learners’ knowledge and confidence surrounding a topic towards a stronger level of understanding [66], to disseminate this focused history script. Because of the interactive nature required of the script, education should include the use of either standardized patients or simulations with learners, followed by a debrief [67,68,69]. Instructional activities can be done either in person or virtually through either synchronous or asynchronous sessions (with video) [70]. This approach should allow learners to transition from information exposure to knowledge and experience, leading to overall increased confidence and cultural proficiency levels. Healthcare professionals have the opportunity, and obligation, to regularly incorporate this script into clinical practice to enhance their cultural proficiency and, simultaneously, their patient care.

### 4.4. Limitations

Our study focused on establishing the content validity of the focused history script. However, future research is needed to establish reliability. We suggest pilot implementation to establish inter-rater reliability, training requirements, and perceived clinical usefulness. As previously addressed, the 40-item script may seem cumbersome to some clinicians. This was accounted for by developing the script to be adaptable in nature to be either a patient-completed questionnaire or to be completed in a one-on-one conversation between clinician and patient. However, identifying potential discrepancies between these different delivery forms has not yet been completed. Further study of this script is needed to gain a more comprehensive understanding of the potential benefits it can provide for providers of the adolescent population. We recommend that research focused on pilot implementation of the script occurs through simulation-specific practice. This method allows for learners to be placed in these experiences to begin the scaffolding learning experience to improve patient-centered care as well as cultural proficiency behaviors, while researchers are simultaneously able to determine the inter-rater reliability of the script objectively. 

## 5. Conclusions

We used a Delphi panel technique through expert consensus to establish content validity for a focused history script to facilitate conversations about SDOH between clinicians and patients within the adolescent population. Incorporation of this script into regular clinical practice will increase cultural proficiency levels displayed by clinicians, leading to a decrease in health disparities caused by SDOH. We suggest implementing the validated script into continuing professional development and professional education programs to promote culturally proficient behaviors of healthcare providers in school-based health centers. Future research is necessary to determine the reliability and clinical usefulness of the script. 

## Figures and Tables

**Table 1:** Panelist Qualifications.

Panelist	Years of Experience	Contemporary Qualifications in SDOH	Work Area(s)	Patient Care Setting
A	4.5	College of public health Master’s degree Research in Healthcare and Social Justice, cultural competency Presenter at various professional conferences	Athletic Trainer	Secondary-Level School-Based Sports Medicine Collegiate-Level School-Based Sports Medicine
B	14	Research in rural & minority health populations, psychosocial determinants of health Recipient of National Institute of Health (NIH) funding for Minority Health and Health Disparities (HHD) HHD grant reviewer	Neurosurgical ICU/Stroke Rehabilitation Unit RN Professor	Hospital (In-patient)
C	18	Research in rural health, SDOH, health policy, health equity Case managing and community organizing focused on housing/mental health	Social services Outpatient psychiatry Professor Rural health research center	Hospital (Outpatient)
D	23	Health Care Alliance Board Member Access to Care Workgroup member for 8+ years Resilience Leader Medical/Dental Sector for New Mexico Part of Adverse Childhood Experiences (ACEs) initiative 2+ years SDOH PPE Event 11+ years	Athletic Trainer PT/AT/OT private practice CEO	Professional Sports Secondary-Level School-Based Sports Medicine Collegiate- Level School-Based Sports Medicine
E	9	Post-doctoral fellowship completing extensive SDOH research New Investigator Grant recipient for SDOH in athletic healthcare Presenter at various professional conferences	Athletic Trainer Professor	Secondary-Level School-Based Sports Medicine Collegiate-Level School-Based Sports Medicine PT clinic
F	26	13+ years of community based coalition work within urban health disparities/obesity initiatives Worksite/university campus wellness Diversity, equity, and inclusion coalition work	Athletic Trainer Community outreach educator Professor	Secondary-Level School-Based Sports Medicine Collegiate-Level School-Based Sports Medicine

**Table 2:** Final Script Item Consensus.

	Reflecting Current Evidence		Script Inclusion		
Item	Mean ± SD	Percent Agreement	Mean ± SD	Percent Agreement	Round Validated:
Do you have a primary care provider, such as a family physician or nurse practitioner? (Y/N)	6.0 ± 0.0	100.0%	5.8 ± 0.8	100.0%	3
How easy is it to access your primary care provider or other medical professionals for your health and well-being?	5.5 ± 0.8	83.0%	5.8 ± 0.4	100.0%	4
How often (annually, for illness only, etc.) do you see your primary care provider?	5.3 ± 1.2	83.3%	5.3 ± 1.2	83.3%	3
Do you have health insurance? (Y/N)	6.0 ± 0.4	100.0%	6.0 ± 0.4	100.0%	4
If not, does that impact whether you go to the doctor or not?	5.0 ± 0.0	100.0%	5.2 ± 0.4	100.0%	5
Was there ever a time where you needed medical care but did not get it?	6.0 ± 0.0	100.0%	6.0 ± 0.0	100.0%	7
If so, why do you think that has happened?	5.2 ± 0.4	100.0%	5.0 ± 0.6	100.0%	5
(If not addressed in the above response) Was cost an issue?	6.0 ± 0.0	100.0%	6.0 ± 0.0	100.0%	7
(If not addressed in the above response) Was quality of care an issue?	5.8 ± 0.4	100.0%	5.8 ± 0.4	100.0%	7
Is it easy to discuss your health with healthcare providers? If not, why?	5.0 ± 1.6	83.3%	5.0 ± 1.6	83.3%	6
Describe how language influences your interactions in healthcare settings.	5.3 ± 0.8	83.3%	5.3 ± 0.8	83.3%	3
What is your family’s level of education?	5.5 ± 0.8	83.3%	5.5 ± 0.8	83.3%	3
What do you expect your next step to be after you finish high school (school, job, military, etc.)?	5.8 ± 0.4	100.0%	5.8 ± 0.4	100.0%	7
Is anything preventing you from achieving any future education plans? If so, can you tell me more?	5.3 ± 0.8	83.3%	5.3 ± 0.8	83.3%	6
How does language influence your interactions in school?	5.2 ± 1.2	83.0%	5.2 ± 1.2	83.0%	4
Branch Question for following sub questions: Recently, or in the past, what concerns, if any, have you had about:					
a. Being evicted (forced out of where you live) or becoming homeless?	5.5 ± 0.8	83.3%	5.3 ± 0.8	83.3%	3
b. Having your power shut off?	5.3 ± 0.8	83.0%	5.5 ± 0.6	100.0%	4
Has your family had any experience worrying about paying monthly bills?	5.7 ± 0.5	100.0%	5.8 ± 0.4	100.0%	4
Has your family had any experience with difficulty affording your clothes and food?	5.8 ± 0.4	100.0%	5.8 ± 0.4	100.0%	4
Was there ever a recreational activity (i.e., sports outside of school teams, musical instrument lessons, etc.) that you wanted to do but couldn’t?	5.7 ± 0.5	100.0%	5.8 ± 0.4	100.0%	7
If yes, was it due to any of the following? (Explain/choose all that apply.) (Cost, Transportation, Scheduling, Other.)	6.0 ± 0.0	100.0%	6.0 ± 0.0	100.0%	7
Describe your living situation.	5.2 ± 0.8	83.3%	5.3 ± 0.8	83.3%	3
Has your family had trouble accessing a grocery store or other markets where you live to get nutritious food?	5.5 ± 0.8	83.0%	5.8 ± 0.4	100.0%	4
Branch Question for following sub questions: Recently, or in the past, what concerns, if any, have you had about:					
a. Having clean running water?	5.5 ± 0.8	83.3%	5.7 ± 0.5	100.0%	3
b. Being exposed to mold or pests where you live?	5.7 ± 0.5	100.0%	5.5 ± 0.6	100.0%	3
c. Feeling safe in your neighborhood?	5.7 ± 0.5	100.0%	5.7 ± 0.5	100.0%	3
What type(s) of transportation do you use most often (car, bus, metro, biking, walking, etc.)?	5.3 ± 0.8	83.3%	5.2 ± 0.8	83.3%	3
If you had an appointment or needed to get somewhere, can you describe how you might get there?	5.7± 0.5	100.0%	5.5 ± 0.8	83.3%	3
Tell me about the parks, sidewalks, and other green spaces that are in your neighborhood.	5.5 ± 0.6	100.0%	5.3 ± 0.8	83.3%	3
Branch Question for following sub questions: Recently, or in the past, what concerns, if any, have you had about:					
a. Abuse in the home (physical, verbal, emotional)?	5.5 ± 0.8	83.0%	5.3 ± 0.5	100.0%	3
b. Family members getting arrested?	5.7 ± 0.5	100.0%	5.3 ± 0.5	100.0%	3
c. Feeling lonely and isolated from your friends and family?	5.5 ± 0.8	83.3%	5.5 ± 0.6	100.0%	3
d. Being discriminated against for any reason?	5.5 ± 0.8	83.3%	5.5 ± 0.6	100.0%	3
Do language barriers affect your ability to talk to people who support you?	5.3 ± 1.2	83.3%	5.7 ± 0.8	83.3%	6
How would you describe your faith, spirituality, or religious beliefs?	5.3 ± 0.8	83.3%	5.3 ± 0.5	100.0%	3
Branch Question for following sub question: For the following questions, think about your experiences with support systems. How is your relationship with the following people:					
a. Your family members/guardians?	5.7 ± 0.8	83.0%	5.7 ± 0.8	83.0%	4
b. Your peers?	5.7 ± 0.8	83.0%	5.7 ± 0.8	83.0%	4
c. Your neighbors?	5.7 ± 0.8	83.0%	5.7 ± 0.8	83.0%	4
d. Adult at school, like teachers or coaches?	5.5 ± 0.8	83.3%	5.5 ± 0.8	83.3%	6
Is there anything I could do to make managing your health easier?	5.3 ± 0.8	83.0%	5.3 ± 0.8	83.0%	5

## Data Availability

Data has been presented in Appendix A, Table A1 of this article.

## Appendix A

**Table A1:** Rounds 3–7 Results.

	Reflecting Current Evidence		Script Inclusion		
Proposed Item	Mean ± SD	Percent Agreement	Mean ± SD	Percent Agreement	Outcome
Round 3 Results.					
Do you have a primary care provider, such as a family physician or nurse practitioner? (Y/N)	6.0 ± 0.0	100.0%	5.8 ± 0.8	100.0%	Validated.
What is your experience with accessing your primary care provider or other medical providers for your health and well-being?	4.8 ± 0.8	66.7%	5.0 ± 0.9	66.7%	Revise to address ease of access.
How often (annually, for illness only, etc.) do you see your primary care provider?	5.3 ± 1.2	83.3%	5.3 ± 1.2	83.3%	Validated.
What is your experience with the healthcare system beyond your primary care provider?	4.8 ± 1.2	66.7%	5.0 ± 0.6	83.3%	Revise to address target population.
Do you have health insurance? (Y/N)	6.0 ± 0.0	100.0%	5.8 ± 0.4	100.0%	Questionnaire error, include in following round.
What is your experience with your use of health insurance?	5.0 ± 0.6	83.3%	5.5 ± 0.6	100.0%	Questionnaire error, include in following round.
What are your experiences with accessing prescription medicine when needed?	4.8 ± 1.7	66.7%	4.5 ± 1.1	50.0%	Revise to be more direct.
Do you use the healthcare system on your own? (Y/N)	5.0 ± 0.9	66.7%	4.7 ± 1.2	50.0%	Eliminated.
If yes: What is your experience using the health system?	4.7 ± 1.0	66.7%	4.7 ± 1.2	50.0%	Eliminated.
What is your experience with understanding topics when discussing health issues or when given resources by healthcare professionals?	5.0 ± 0.9	66.7%	5.2 ± 1.0	66.7%	Revise to be more conversational.
Describe how language influences your interactions in healthcare settings.	5.3 ± 0.8	83.3%	5.3 ± 0.8	83.3%	Validated.
Tell me a little about your family’s education.	4.8 ± 1.3	66.7%	5.0 ± 0.6	83.3%	Revise to address those living with the patient.
What is your family’s level of education?	5.5 ± 0.8	83.3%	5.5 ± 0.8	83.3%	Validated.
What is your experience with attending school?	4.7 ± 1.0	66.7%	4.7 ± 0.8	50.0%	Revise to address education quality.
What are your thoughts and plans for any educational needs you may have for the future?	5.2 ± 1.2	83.3%	5.0 ± 0.9	66.7%	Revise to be more conversational.
Is anything preventing you from additional educational opportunities? If so, please elaborate.	5.5 ± 0.8	83.3%	5.2 ± 1.0	66.7%	No revisions to be made—argument: Education has a direct impact on a person’s health, so educational barriers can lead to a variety of factors decreasing health. Refer to citation.
Describe how language influences your interactions in school.	5.2 ± 1.0	66.7%	5.0 ± 1.3	66.7%	Revise to be more conversational.
Describe your financial situation.	5.0 ± 1.3	66.7%	5.0 ± 1.6	83.3%	No revisions to be made—argument: Item is intended to explore the socioeconomic status of the patient.
Branch Question for following sub questions: Recently, or in the past, what concerns, if any, have you had about:					
a. Being evicted (forced out of where you live) or becoming homeless?	5.5 ± 0.8	83.3%	5.3 ± 0.8	83.3%	Validated.
b. Having your power shut off?	5.2 ± 1.0	66.7%	5.0 ± 0.9	66.7%	No revisions to be made—argument: Various existing SDOH screening tools address concerns of lack of power, indicating the importance of keeping this item in the script.
What is your family’s experience with paying monthly bills?	5.3 ± 0.8	83.3%	5.0 ± 1.3	66.7%	Revise to be more conversational.
What is your family’s history with affording your clothes and food?	5.3 ± 0.8	83.3%	5.0 ± 1.1	50.0%	Revise to be more conversational.
What is your family’s experience with affording recreational activities? (i.e., Sports outside of school teams, musical instrument lessons, etc.).	5.2 ± 1.0	66.7%	5.2 ± 1.2	83.3%	Revise to be more conversational.
Describe your living situation.	5.2 ± 0.8	83.3%	5.3 ± 0.8	83.3%	Validated.
What is your experience with accessing a grocery store or other markets where you live to get nutritious food?	5.3 ± 0.8	83.3%	5.2 ± 1.0	66.7%	Revise to be more conversational.
Branch Question for following sub questions: Recently, or in the past, what concerns, if any, have you had about:					
a. Having clean running water?	5.5 ± 0.8	83.3%	5.7 ± 0.5	100.0%	Validated.
b. Being exposed to mold or pests where you live?	5.7 ± 0.5	100.0%	5.5 ± 0.6	100.0%	Validated.
c. Feeling safe in your neighborhood?	5.7 ± 0.5	100.0%	5.7 ± 0.5	100.0%	Validated.
What type(s) of transportation do you use most often (car, bus, metro, biking, walking, etc.)?	5.3 ± 0.8	83.3%	5.2 ± 0.8	83.3%	Validated.
If you had an appointment or needed to get somewhere, can you describe how you might get there?	5.7 ± 0.5	100.0%	5.5 ± 0.8	83.3%	Validated.
Tell me about the parks, sidewalks, and other green spaces that are in your neighborhood.	5.5 ± 0.6	100%	5.3 ± 0.8	83.3%	Validated.
Branch Question for following sub questions: Recently, or in the past, what concerns, if any, have you had about:					
a. Abuse in the home (physical, verbal, emotional)?	5.5 ± 0.8	83.0%	5.3 ± 0.5	100.0%	Validated.
b. Family members getting arrested?	5.7 ± 0.5	100.0%	5.3 ± 0.5	100.0%	Validated.
c. Feeling lonely and isolated from your friends and family?	5.5 ± 0.8	83.3%	5.5 ± 0.6	100.0%	Validated.
d. Being discriminated against for any reason?	5.5 ± 0.8	83.3%	5.5 ± 0.6	100.0%	Validated.
How would you describe your faith, spirituality, or religious beliefs?	5.3 ± 0.8	83.3%	5.3 ± 0.5	100.0%	Validated.
Describe how language influences your interactions in social settings.	5.3 ± 1.0	66.7%	5.3 ± 0.8	83.3%	Revise to be more conversational.
Branch Question for following sub questions: For the following questions, think about your experiences with support systems:					
a. Describe how your relationship is with your family members/guardians.	5.5 ± 0.8	83.3%	5.7 ± 0.5	100.0%	Revise to improve question flow and structure.
b. Describe your relationship with your peers.	5.3 ± 0.8	83.3%	5.5 ± 0.6	100.0%	Validated*
c. Describe your relationship with your neighbors.	5.3 ± 0.8	83.3%	5.2 ± 1.0	66.7%	Revise to improve question flow and structure.
d. Describe your relationship with your teachers/coaches/other adult figures.	5.2 ± 1.0	66.67%	5.2 ± 1.0	66.7%	Revise to improve question flow and structure, and to be more conversational.
e. Describe your relationship with healthcare professionals.	4.5 ± 1.6	50.0%	4.7 ± 1.2	50.0%	Revise to improve question flow and structure, and to be more conversational.
Round 4 Results.					
How easy is it to access your primary care provider or other medical professionals for your health and well-being?	5.5 ± 0.8	83.0%	5.8 ± 0.4	100.0%	Validated.
How easy is it to navigate the healthcare system? On your own?	4.7 ± 0.8	83.0%	5.2 ± 1.0	67.0%	Revise to a singular question.
Do you have health insurance? (Y/N)	6.0 ± 0.4	100.0%	6.0 ± 0.4	100.0%	Validated.
What is your experience with your use of health insurance?	4.8 ± 1.2	67.0%	5.3 ± 1.0	67.0%	Revise to a sub-question of previous item.
Have you ever gone without medication or medical care due to concerns of cost?	5.3 ± 1.0	67.0%	5.7 ± 0.5	100.0%	Revise to be more open-ended, with a follow up sub-question.
How well do you understand topics when discussing health issues or when given resources by healthcare professionals?	5.2 ± 1.0	67.0%	5.5 ± 0.8	83.0%	Revise to be more conversational.
Tell me a little about the education of relatives or those who live with you.	5.0 ± 1.3	67.0%	5.2 ± 1.0	67.0%	Eliminated.
How do you feel about the quality of schooling you have received?	5.4 ± 1.0	67.0%	5.4 ± 1.0	67.0%	Revise to be more conversational.
What are your thoughts and plans for your education in the future?	4.8 ± 1.2	67.0%	4.8 ± 1.2	67.0%	Revise to be more inclusive of all career paths post-secondary school completion.
Is anything preventing you from additional educational opportunities? If so, please elaborate.	5.3 ± 1.0	67.0%	5.3 ± 1.0	67.0%	Revise to be more conversational.
How does language influence your interactions in school?	5.2 ± 1.2	83.0%	5.2 ± 1.2	83.0%	Validated.
Describe your financial situation.	4.8 ± 1.3	50.0%	4.8 ± 1.1	33.0%	Eliminated.
Branch Question for following sub question: Recently, or in the past, what concerns, if any, have you had about:					
b. Having your power shut off?	5.3 ± 0.8	83.0%	5.5 ± 0.6	100.00%	Validated.
Has your family had any experience worrying about paying monthly bills?	5.7 ± 0.5	100.0%	5.8 ± 0.4	100.00%	Validated.
Has your family had any experience with difficulty affording your clothes and food?	5.8 ± 0.4	100.0%	5.8 ± 0.4	100.0%	Validated.
Has your family had any experience with difficulty affording recreational activities? (i.e., Sports outside of school teams, musical instrument lessons, etc.)	5.3 ± 1.0	67.0%	5.2 ± 1.0	67.0%	No revisions to be made—argument: Recreation/sport activity contributes to enhancement of quality of life.
Has your family had trouble accessing a grocery store or other markets where you live to get nutritious food?	5.5 ± 0.8	83.0%	5.8 ± 0.4	100.0%	Validated.
How does language influence your interactions in social settings?	5.5 ± 0.8	83.0%	5.3 ± 1.0	67.0%	Revise to be more conversational and more reflective of question intention.
Branch Question for following sub questions: For the following questions, think about your experiences with support systems. How is your relationship with the following people:					
a. Your family members/guardians?	5.7 ± 0.8	83.0%	5.7 ± 0.8	83.0%	Validated.
b. Your peers?	5.7 ± 0.8	83.0%	5.7 ± 0.8	83.0%	Validated.
c. Your neighbors?	5.7 ± 0.8	83.0%	5.7 ± 0.8	83.0%	Validated.
d. Your teachers/coaches/other adult figures?	5.3 ± 0.8	83.0%	5.2 ± 1.1	50.0%	Revise to be more conversational.
e. Your athletic trainer?	5.3 ± 0.8	83.0%	5.2 ± 1.0	83.0%	Revise to be more quality improvement focused.
Round 5 Results.					
How easy is it to navigate the healthcare system on your own?	5.2 ± 1.0	67.0%	5.2 ± 1.0	67.0%	Revise to be more conversational and address target population.
If not, does that impact whether you go to the doctor or not?	5.0 ± 0.0	100.0%	5.2 ± 0.4	100.0%	Validated.
Have you ever felt like you’ve gone without medical care?	5.0 ± 0.8	67.0%	5.3 ± 0.8	83.0%	No revisions made— argument: avoidance of healthcare can be an indicator of lack of access.
If so, why do you think that has happened?	5.2 ± 0.4	100.0%	5.0 ± 0.6	100.0%	Validated.
How easy is it to talk about your health with healthcare providers?	5.0 ± 0.9	67.0%	5.3 ± 0.8	83.0%	Revise to be more direct, with a follow up sub-question.
Are you satisfied with the quality of schooling you’ve had? If not, can you explain?	4.7 ± 1.2	50.0%	5.0 ± 0.9	67.0%	Eliminated.
Do you have thoughts and plans for any future education? If so, can you explain?	4.8 ± 1.2	67.0%	5.0 ± 0.9	67.0%	Revise to be more conversational.
Is anything preventing you from achieving your future education? If so, can you tell me more?	5.3 ± 1.0	67.0%	5.3 ± 1.0	67.0%	No revisions made— argument: higher education has a direct impact on health.
Has your family had any experience with difficulty affording recreational activities (i.e., sports outside of school teams, musical instrument lessons, etc.)?	5.3 ± 1.2	83.0%	5.2 ± 1.0	67.0%	Revise to be more conversational.
How does language influence your interactions in different social support systems?	4.7 ± 1.0	67.0%	5.0 ± 0.9	67.0%	Revise to be more specific.
Branch Question for following sub question: For the following questions, think about your experiences with support systems. How is your relationship with the following people:					
d. Adult figures at school?	4.5 ± 0.9	67.0%	4.45 ± 0.9	67.0%	Revise to provide examples.
Is there anything I could do to make managing your health easier?	5.3 ± 0.8	83.0%	5.3 ± 0.8	83.0%	Validated.
Round 6 Results.					
Have you ever felt like you’ve gone without medical care?	5.2 ± 1.6	83.3%	5.2 ± 1.3	66.7%	Revise to be more specific.
(If not addressed in the above response) Was cost an issue? Was quality of care an issue?	5.6 ± 0.6	83.3%	5.0 ± 1.3	66.7%	Revise to multiple sub-questions.
Is it easy to discuss your health with healthcare providers? If not, why?	5.0 ± 1.6	83.3%	5.0 ± 1.6	83.3%	Validated.
What are you thinking of doing after you finish high school?	5.0 ± 1.7	66.7%	5.0 ± 1.7	66.7%	Revise to provide examples.
Is anything preventing you from achieving any future education plans? If so, can you tell me more?	5.3 ± 0.8	83.3%	5.3 ± 0.8	83.3%	Validated.
Has your family had difficulty affording recreational activities? (i.e., sports outside of school teams, musical instrument lessons, etc.)	5.3 ± 1.0	66.7%	5.3 ± 1.0	66.7%	Revise to be more specific, with follow up sub-questions addressing specific reasons.
Do language barriers affect your ability to talk to people who support you?	5.3 ± 1.2	83.3%	5.7 ± 0.8	83.3%	Validated.
Branch Question for following sub question: For the following questions, think about your experiences with support systems. How is your relationship with the following people:					
d. Adults at school, like teachers or coaches?	5.5 ± 0.8	83.3%	5.5 ± 0.8	83.3%	Validated.
Round 7 Results.					
Was there ever a time where you needed medical care but did not get it?	6.0 ± 0.0	100.0%	6.0 ± 0.0	100.0%	Validated.
(If not addressed in the above response) Was cost an issue?	6.0 ± 0.0	100.0%	6.0 ± 0.0	100.0%	Validated.
(If not addressed in the above response) Was quality of care an issue?	5.8 ± 0.4	100.0%	5.8 ± 0.4	100.0%	Validated.
What do you expect your next step to be after you finish high school (school, job, military, etc.)?	5.8 ± 0.4	100.0%	5.8 ± 0.4	100.0%	Validated.
Was there ever a recreational activity (i.e., sports outside of school teams, musical instrument lessons, etc.) that you wanted to do but couldn’t?	5.7 ± 0.5	100.0%	5.8 ± 0.4	100.0%	Validated.
If yes, was it due to any of the following? (Explain/choose all that apply.) (Cost, Transportation, Scheduling, Other.)	6.0 ± 0.0	100.0%	6.0 ± 0.0	100.0%	Validated.

* as question flow and structure needs to be revised, item must be re-validated.
